# Operation for Adiposity

**Published:** 1901-06-22

**Authors:** 


### OPERATION FOR ADIPOSITY.

AN interesting case is reported by Dr. Lindsay Peters, in the "Annals of Surgery" for March, in which he removed a large sheet of adipose tissue from the front of the abdominal muscles for the relief of a pendulous condition of the abdomen. The patient was a woman, 32 years old, who had not been unduly stout until about ten years before, when her breasts became so large and pendulous that after a few years she had them removed, when they were found to weigh nearly 25 lb. Then fat began to accumulate in the abdominal walls, which hung down in large folds reaching nearly to the knees when she sat down. An enormous crescent of abdominal skin and subcutaneous fat was removed by operation. The patient being placed on her back, a tranverse incision was made across the upper part of the abdomen, extending from the point of contact with the table on one side to a similar spot on the opposite side; the fat was separated from the underlying muscles and peeled downwards, skin and all, and a lower transverse incision was made a little above the line of reflexion of the pendulous fold, the two incisions meeting at their extremities. Everything between these incisions was then removed down to the muscles, and the wound was closed by silkworm and catgut sutures. The removed mass weighed 7,450 grammes, or over 15 lb.; but when she got up, a month after the operation, it was found that she had lost a good deal more weight than that. She had had transient glycosuria, but it passed away after the operation.

